# Preoperative OCT Characteristics Contributing to Prediction of Postoperative Visual Acuity in Eyes with Macular Hole

**DOI:** 10.3390/jcm13164826

**Published:** 2024-08-15

**Authors:** Yoko Mase, Yoshitsugu Matsui, Koki Imai, Kazuya Imamura, Akiko Irie-Ota, Shinichiro Chujo, Hisashi Matsubara, Hiroharu Kawanaka, Mineo Kondo

**Affiliations:** 1 Department of Ophthalmology, Mie University Graduate School of Medicine, Tsu 514-8507, Mie, Japan; 2 Department of Electrical and Electronic Engineering, Mie University, Tsu 514-8507, Mie, Japan

**Keywords:** optical coherence tomography, macular hole, logistic regression, machine learning, clinical prediction models

## Abstract

**Objectives:** To develop a machine learning logistic regression algorithm that can classify patients with an idiopathic macular hole (IMH) into those with good or poor vison at 6 months after a vitrectomy. In addition, to determine its accuracy and the contribution of the preoperative OCT characteristics to the algorithm. **Methods:** This was a single-center, cohort study. The classifier was developed using preoperative clinical information and the optical coherence tomographic (OCT) findings of 43 eyes of 43 patients who had undergone a vitrectomy. The explanatory variables were selected using a filtering method based on statistical significance and variance inflation factor (VIF) values, and the objective variable was the best-corrected visual acuity (BCVA) at 6 months postoperation. The discrimination threshold of the BCVA was the 0.15 logarithm of the minimum angle of the resolution (logMAR) units. **Results:** The performance of the classifier was 0.92 for accuracy, 0.73 for recall, 0.60 for precision, 0.74 for F-score, and 0.84 for the area under the curve (AUC). In logistic regression, the standard regression coefficients were 0.28 for preoperative BCVA, 0.13 for outer nuclear layer defect length (ONL_DL), −0.21 for outer plexiform layer defect length (OPL_DL) − (ONL_DL), and −0.17 for (OPL_DL)/(ONL_DL). In the IMH form, a stenosis pattern with a narrowing from the OPL to the ONL of the MH had a significant effect on the postoperative BCVA at 6 months. **Conclusions:** Our results indicate that (OPL_DL) − (ONL_DL) had a similar contribution to preoperative visual acuity in predicting the postoperative visual acuity. This model had a strong performance, suggesting that the preoperative visual acuity and MH characteristics in the OCT images were crucial in forecasting the postoperative visual acuity in IMH patients. Thus, it can be used to classify MH patients into groups with good or poor postoperative visual acuity, and the classification was comparable to that of previous studies using deep learning.

## 1. Introduction

A macular hole (MH), typically idiopathic, is caused by an abnormal vitreomacular traction. It is a common ophthalmic abnormality that can rapidly progress to a reduction of the visual acuity. There have been significant advances in the diagnosis and treatment of MHs. The advanced surgical techniques include pars plana vitrectomy (PPV) and internal limiting membrane (ILM) peeling with or without an inverted ILM flap. The evolution of these diagnostic methods and treatments has been driven by advances in imaging technology, especially over the past 20 years. These advances have led to impressive advances in the interpretation of OCT images, and the ability to evaluate pathological conditions and disease progression using cross-sectional retinal images [1]. However, predicting the postoperative visual acuity remains difficult even with a surgical closure rate of 85%. The postoperative visual acuity is influenced by many factors associated with the MH, such as its preoperative minimum linear diameter, the MH index, the basal diameter, and the stage of the MH [2,3,4,5,6]. However, the causal relationships and the correlations are complicated.

Machine learning (ML) is a tool that can be trained to predict a subjective variable using explanatory variables that are statistically significant. It can analyze complex relationships between multiple variables. Relevant to this study, the application of ML in a Medical Decision Support System (MDSS) can enhance the quality of patient care by offering clinicians relevant information based on the patient’s clinical characteristics. This provides a secondary and objective perspective to aid in the medical decision-making processes [7,8].

Our group has published the results of using a nonlinear ML algorithm to predict the BCVA in eyes with a macular edema (ME) due to a branch retinal vein occlusion (BRVO) during the first-year maintenance phase of anti-VEGF therapy [9]. The clinical information and OCT findings obtained at the first resolution of the ME after the initial treatment were used in that study. We have also shown that a linear algorithm can predict the BCVA in the same patients with good accuracy, and we have determined the accuracy of the linear classifiers and the degree of contribution by the explanatory variables [10].

Although there have been studies on the anatomic closure prognosis using the preoperative OCT features by ML for MHs [11,12], there are only two studies that examined the postoperative visual outcomes using artificial intelligence (AI) algorithms [13,14]. Both of these studies used deep learning (DL) for the preoperative OCT images, but these techniques had limited explanatory potential for prediction due to their ‘black-box’ nature.

Thus, the purpose of this study was to examine MH patients using ML, similar to the methods used in the BRVO study, to improve the predictive performance and to interpret the complex interplay of multiple factors of the OCT findings. More specifically, we aimed to determine the contribution of each explanatory variable on the classification and prediction of individual postoperative visual outcomes after a vitrectomy. This was accomplished by establishing single-label, two-class problems to categorize patients into Group A with good visual outcomes and Group B with poor visual outcomes. In addition, we determined the accuracy of the linear classifiers and the degree of contribution of the explanatory variables to the algorithm.

## 2. Materials and Methods

### 2.1. Patients and Treatment

This was a single-center, cohort study of the medical records of patients examined in the Department of Ophthalmology, Mie University Hospital, between January 2017 and October 2020 (ethical approval code: H2019-219). To create this prediction model, we followed the TRIPOD checklist [15].

Patients with a MH who had undergone surgery with at least 6 months of follow-up examinations were studied. The inclusion criteria were patients whose reduced visual acuity was solely due to the MH, an absence of concurrent retinal diseases, and no missing data of the explanatory variables. The exclusion criteria were cases of reoperation and cases where there was a failure to close the MH. All patients had undergone either a vitrectomy or phacovitrectomy, ILM peeling, and gas tamponade with gas as needed (2 with Air, 1 with C3F8, and the others with SF6) with several days of postoperative overhead bed rest at the same institution.

### 2.2. Dividing Patients into Two Groups Based on the Best-Corrected Visual Acuity

The best-corrected visual acuity (BCVA) was recorded at the baseline and at each examination following the treatment. The primary outcomes were the BCVA at 6 months post-operation. We defined Group A (good prognosis) as patients achieving a decimal BCVA of ≥0.7, and Group B (poor prognosis) consisted of patients with decimal BCVA of <0.7. A threshold of 0.15 logMAR units (approximate decimal BCVA of 0.7) was used, as this is the visual acuity required for the renewal of a Japanese driver’s license and reflects the level of visual acuity desired by patients in a clinical setting.

### 2.3. Preprocessing of OCT Images and Features

Spectral domain (SD) optical coherence tomographic (OCT) images were recorded with the Spectralis OCT device (Spectralis HRA + OCT, Heidelberg Engineering, Heidelberg, Germany) at the baseline, and at the 1, 3, and 6 months postoperative examinations.

**Figure 1:**
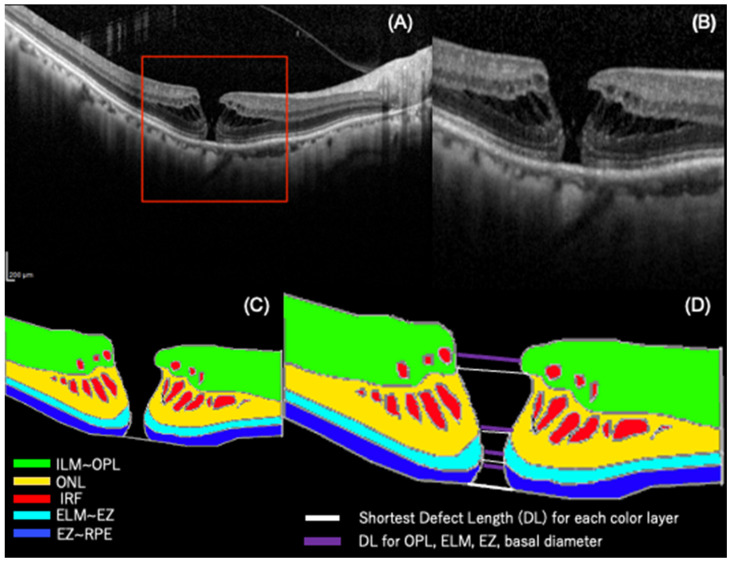
Preprocessing and annotation of OCT images to extract explanatory variables. (**A**) A preoperative optical coherence tomographic (OCT) image of an eye with a macular hole (MH). (**B**) Trimmed image of the OCT image in the red frame in (**A**). (**C**) Annotation for the outer plexiform (OPL) line, external limiting membrane (ELM) line, ellipsoid zone (EZ) line, and retinal pigment epithelial (RPE) line as demarcation lines in a trimmed image. The top layer of the image was painted green then in yellow, sky-blue, and blue in that order. The intraretinal fluid (IRF) was painted red. (**D**) The Shortest Defect Length (DL) for each color layer and DL for the OPL, ELM, EZ, and basal diameter.

Instead of using the baseline OCT image as a feature, we annotated the OCT image and used the raster and vector data in that region as handcrafted features. The original OCT image size was 768 × 496 pixels (9 × 1.9 mm, 30°), and it was cropped to create a 256 × 256 pixels (0.98 × 0.98 mm, 3.26°) trimmed image. The trimming process was completed manually by an examiner (YM) with the outer retinal layer of the fovea as the center of the trimmed image (Figure 1A).

In the cropped images, the vector and raster data were uniquely defined as handcrafted features in the structural regions related to the visual function of the macular hole, as in earlier studies [2,3,4,16,17]. The outer plexiform layer (OPL), external limiting membrane (ELM), ellipsoid zone (EZ), intraretinal fluid (IRF), and retinal pigment epithelium (RPE) were manually marked and highlighted on the trimmed images using the GNU Image Manipulation Program (GIMP) (v2.10.38), an image editing software (Figure 1C) [18].

This process yielded nine types of handcrafted features for objective evaluations. They included the following: (1) inter-edge distance for layers such as the OPL, ELM, and EZ, (2) minimum inter-layer distance (green, yellow, sky-blue, blue), and (3) minimum linear diameter and base diameter (Figure 1D). A total of 41 features were established from the patients’ background information and features of the OCT images (Table 1). To ensure consistency and accuracy, the manual annotations of the OCT images were performed by a single retina specialist and subsequently reviewed by another retina specialist.

### 2.4. Machine Learning Algorithms Considering AI Alignment

**Figure 2:**
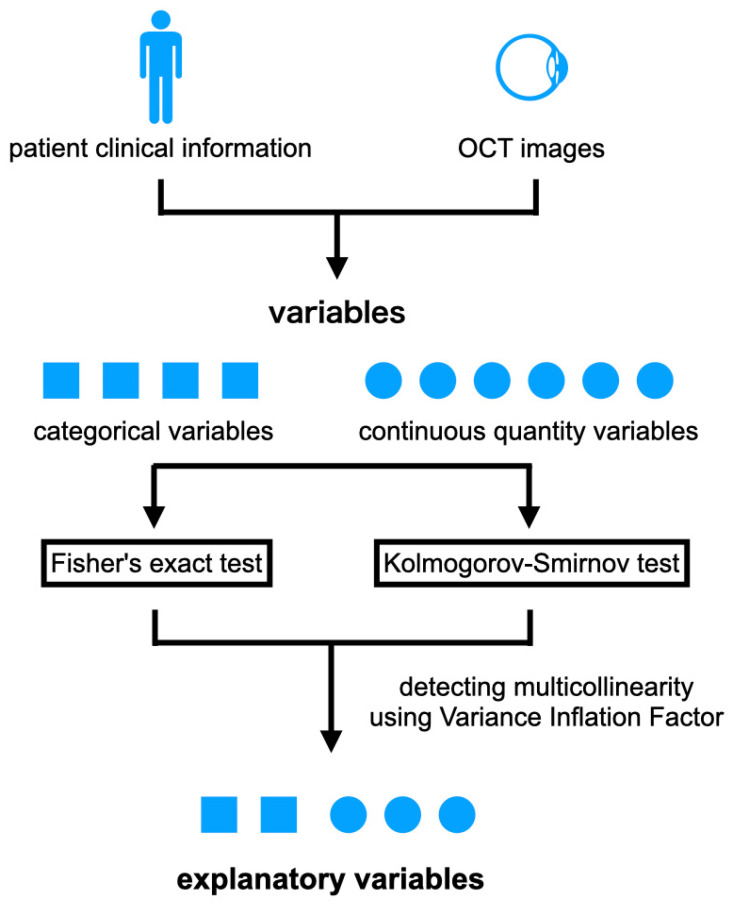
Fisher’s probability of correct answer tests were performed for the categorical variables extracted from the clinical information and OCT images. Kolmogorov–Smirnov tests were performed for continuous quantitative variables. Variables that had a statistically significant difference between Group A and Group B were used as candidates for the explanatory variables. Among these variables, those with a variance inflation factor values > 10, i.e., with R^2^ scores > 0.9, were eliminated, and the tests were repeated until the R^2^ score for all variables was <0.9. Finally, the remaining variables were used as the explanatory variables.

Logistic regression was used for the classifiers [19]. Features with statistically significant differences between the two groups served as the explanatory variables (Figure 2). A stratified cross-validation method for both test and parameter tuning was used so that the ratio of Group A to Group B was the same for each block when the data were divided. This resulted in dividing the dataset into five blocks [20]. One block was used for the test data and four blocks were used for the training data. For the test data, 100 pairs were randomly selected from all combinations of patients by performing 5 stratified cross-validation 100 times.

**Figure 3:**
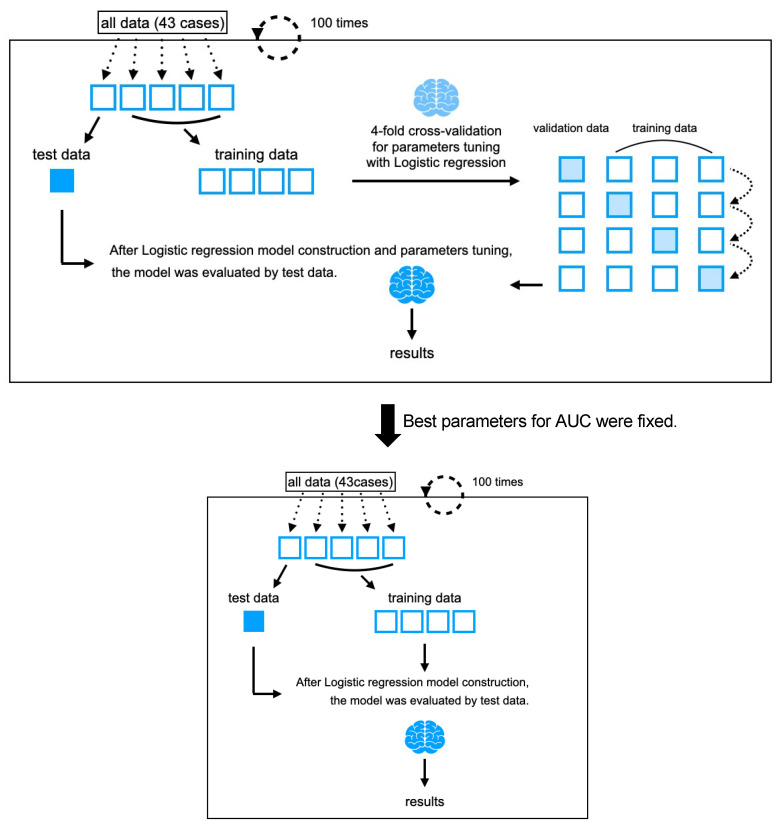
The algorithm shown in the upper box was used to adjust the parameters to maximize the area under the curve (AUC). Then, as shown in the algorithm in the lower box, a logistic regression model was created with the training data by fixing the parameters at the most frequent values among those explored in the upper box, and then the model was evaluated with the test data.

The AI alignment of the classifiers to predict the patient’s visual function prior to treatment was carefully considered in terms of not compromising the patient’s motivation for treatment [21]. For the adjusted classification thresholds, we adjusted the parameters of the algorithm so that the accuracy was higher than the recall. The tuning of the parameter was adjusted to maximize their accuracy (Figure 3).

The accuracy, precision, recall, and F-measure were used to evaluate the cross-validation, and the interval estimation was also determined. The contribution of the explanatory variables to the predictions was assessed using standard regression coefficients. These tasks were implemented using the Python library “scikit-learn” [22].

### 2.5. Statistical Analyses

The explanatory variables were selected by comparing the features between the two groups and using the following tests. The Kolmogorov–Smirnov test was used for continuous quantitative variables. Fisher’s exact test was used as a categorical variable. The Wilcoxon signed-rank test was also used to test the association between the preoperative BCVA and the 6-month BCVA. Each variable was considered statistically significant when *p* < 0.05. All statistical analyses were performed using the statistical programming language R (R version 3.1.3; e foundation for Statistical Computing, Vienna, Austria).

## 3. Results

### 3.1. Demographics of Group A and Group B

In total, 43 eyes of 43 patients met the eligibility criteria, and 32 eyes were placed in Group A and 11 eyes in Group B. The demographics of each group are presented in Table 2. Group A has a mean age of 65.6 years, whereas Group B has a mean age of 67.2 years. The male proportion is 30.3% in Group A and 36.4% in Group B. The majority of patients underwent phacovitrectomy procedures. Comparisons of the two groups showed that only the preoperative BCVA was statistically different between Groups A (BCVA = 0.46) and B (BCVA = 0.81).

### 3.2. Explanatory Variables

From 32 handcraft features extracted from the OCT images and 9 features obtained from the patients’ clinical information, significant differences between Group A and Group B were observed in 5 features (Table 2). The R^2^ values were calculated to exclude features with multicollinearity (Table 3). There were multicollinearities between the features that were statistically different between Groups A and B. These included the ELM defect length (DL), which was excluded, and the other features were selected as explanatory variables.

### 3.3. Hyperparameters of Logistic Regression

**Figure 4:**
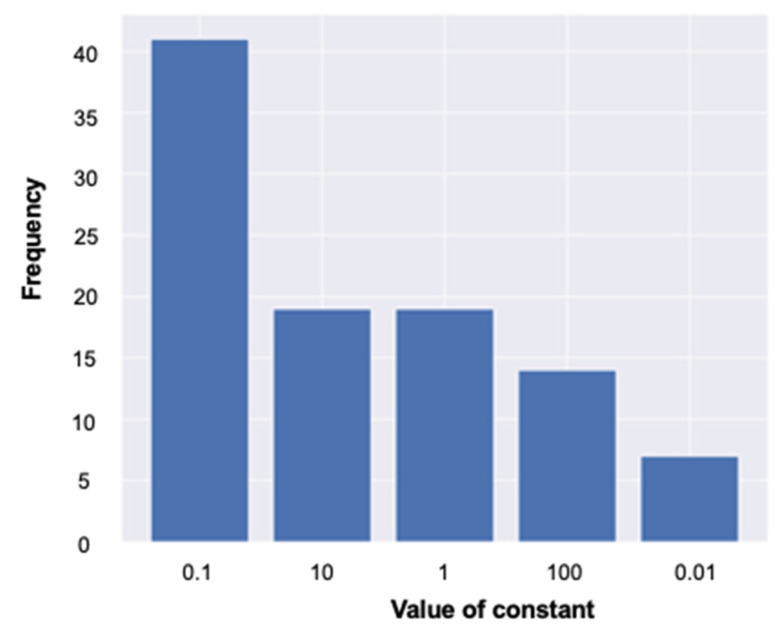
The frequency of each of the constant terms of the parameters is shown.

The frequency of the occurrence of hyperparameters in 100 pre-tests for the adjustments of the hyperparameters with an F1 score is shown in Figure 4. Based on this, the hyperparameter used in the final test was fixed with a constant of 0.1.

### 3.4. Classification Performance

**Figure 5:**
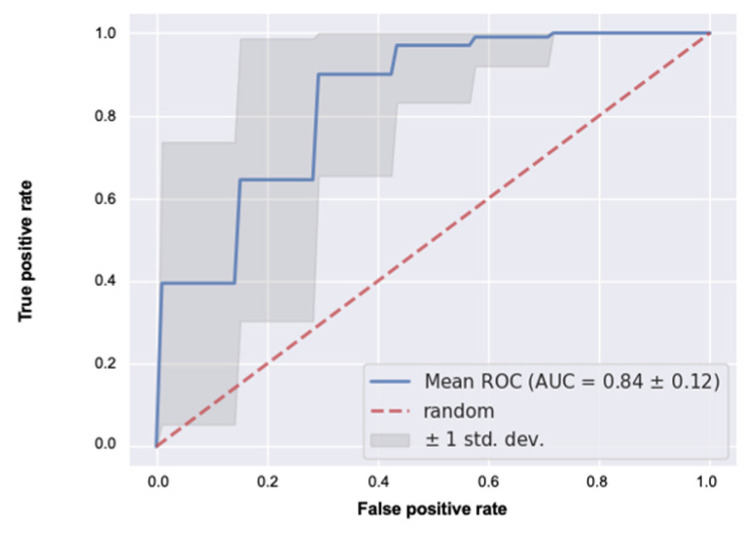
The mean receiver operating characteristic (ROC) curve of the classifiers for Group A and Group B at 6 months postoperation. The standard deviation (SD) is equivalent to the SD of the area under the ROC curve (AUC) obtained by evaluating the model 100 times with the test data.

The predictive performance of the classification between Groups A and B is shown in Table 4 and Figure 5. The precision was 0.92, recall was 0.73, accuracy was 0.74, F-score was 0.80, and AUC was 0.84.

### 3.5. Specific Contributions of Explanatory Variables

**Figure 6:**
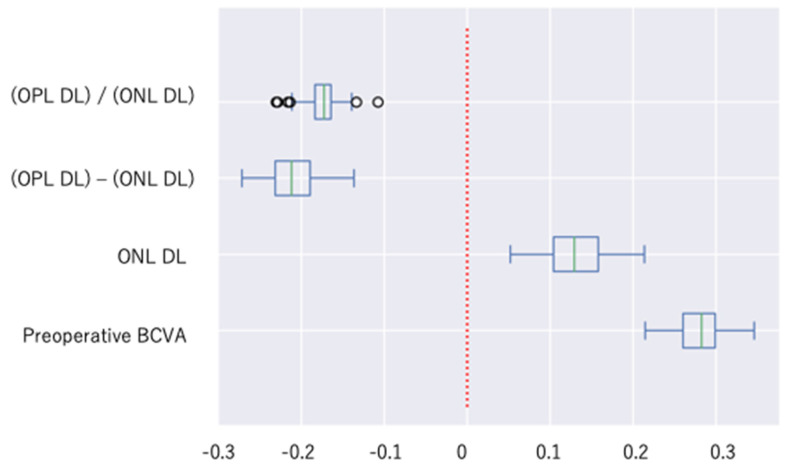
Box-and-whisker diagrams with outliers showing coefficients of the explanatory variables at 6 months postoperation. The maximum and minimum whisker lengths are set at the upper and lower limits of 1.5 times the interquartile range (IQR). The first quartile −1.5 × IQR is the lower limit of the whisker, and the third quartile +1.5 × IQR is the upper limit of the whisker. Values less than the lower end of the whisker or more than the upper end of the whisker are indicated by circles as outliers.

The regression coefficients determined are shown in Figure 6. The regression coefficients of the explanatory variables were as follows: preoperative BCVA was 0.281, ONL_DL was 0.130, (OPL_DL)/(ONL_DL) was −0.174, and (OPL_DL) − (ONL_DL) was −0.212 (Table 5).

### 3.6. Control Experiment

**Figure 7:**
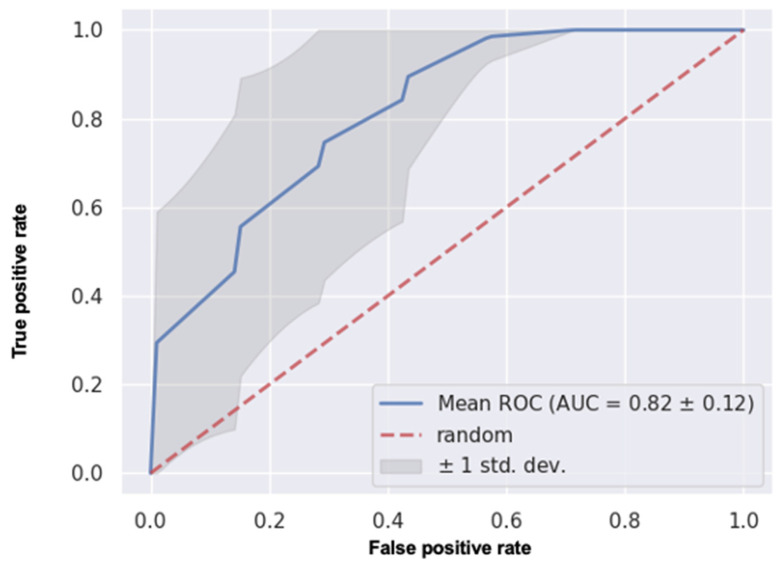
The mean receiver operating characteristic (ROC) curve of the classifiers for Group A and Group B at 6 months postoperation using only the patients’ clinical information. The standard deviation (SD) is equivalent to the SD of the area under the ROC curve (AUC) obtained by evaluating the model 100 times with the test data.

In a control experiment using only logistic regression on the nine features obtained from the patients’ clinical information, significant differences were observed for the preoperative BCVA between Groups A and B. The hyperparameters used in the final test were fixed with a constant of 0.1. The precision was 0.863, recall was 0.764, accuracy was 0.715, F-score was 0.802, and AUC was 0.82 ± 0.12 (Figure 7).

## 4. Discussion

The methods used in our earlier study on BRVO were used in this study on MHs, which was intended to determine the individual contributions of each explanatory variable in classifying and predicting the postoperative BCVA [9,10]. A vitrectomy for MHs has improved the postoperative visual acuity, and the predicted AUC at 6 months postoperation during the recovery process was 0.84 ± 0.12.

We also created a high precision/low recall model aimed at encouraging patients to pursue treatment. At 6 months postoperation, the precision value was 0.92 and the recall value was 0.73. These values agree with our goals.

In a control experiment where the OCT features were removed from the explanatory variables, the AUC was 0.82 ± 0.12 (Figure 7), and the OCT features contributed only slightly to the improvement in the prediction of the postoperative BCVA in eyes with a MH.

ML was chosen over DL due to the small sample size and concerns about over-training leading to a poor generalizability performance and low explainability [23].

Previous reports on the predictions of the postoperative visual acuity have mainly used DL and not ML. For example, Lachance et al. employed a “hybrid regression model”, which integrated clinical features with convolutional neural networks’ (CNNs’) predictions, and achieved an AUC of 0.81 ± 0.05. These findings indicated that, although the inclusion of OCT images slightly enhanced the preoperative data, its impact was minimal. Similarly, Obata et al. used the preoperative information and OCT images, and they reported a superior prediction outcome based on the OCT images alone, as compared to that including the preoperative patient information. However, the accuracy was similarly moderate at 46% vs. 40% [13,14]. The minimal impact of OCT images on preoperative data, as noted by these studies, may be attributed to the overlapping information obtained from both the clinical and OCT features. Our findings are consistent with the reports of Lachance et al. and Obata et al., which also highlight the importance of preoperative visual acuity. The predictive performance of our study is comparable to them. However, our study uniquely identified the significance of specific OCT-derived metrics that offered new insights into the predictors of the visual outcomes.

One strength of deep learning (DL) is its ability to process large datasets through direct image processing that improves the accuracy but reduces the interpretability. In contrast, ML can detect the relevant areas of the OCT images for evaluation that contribute to the postoperative predictions. This can then provide valuable information for understanding the pathological conditions in OCT research. The accuracy of the predictions was comparable to that reported by Lachance et al. [13]. In our study, the OCT images could not be used because this was not DL, and based on the prior literature, the features were designed by manually annotating the stratified structures and then calculating the length and area values as vector data. Obata et al. analyzed subsets of patients with inaccurate predictions and were unable to determine the causes due to the limited interpretability. In contrast, our ML approach allowed us to determine the contribution of each explanatory variable. The following section describes the characteristics of the explanatory variables that significantly contributed to our predictions.

Although the results are limited to the data used in this study, the originally defined explanatory variables for the combination of vector data focusing on the layer structure of OCT images were more effective in predicting than the reported explanatory variables contributing to the visual function.

There have been many reports on the preoperative OCT morphology and postoperative visual prognosis or anatomy of the MHs. Specifically, there is vector data information for distances such as the basal diameter, minimum linear diameter, ELM_DL, and EZ_DL [24,25,26,27]. There is also the diameter hole index (DHI) and the hole diameter ratio (HDR), which are combinations of the vector data information.

We used the filter method to select the explanatory variables from a stratified distance, minimum linear diameter, base diameter, ELM_DL, EZ_DL, stratified area, IRF area, and their combinations as features and examined the explanatory variables that contributed to the prediction.

We found that a poor preoperative BCVA was associated with a poor prognosis at 6 months postoperation, while a larger (OPL_DL) − (ONL_DL) and (OPL_DL)/(ONL_DL), and longer ONL_DL contributed to a good prognosis for the BCVA at 6 months postoperation.

In comparison with earlier reports, our study showed that poor preoperative visual acuity contributed to a poorer visual acuity at 6 months in our study, as well as in many other reports [28,29]. Furthermore, our study uniquely identified the significance of specific OCT-derived metrics, offering new insights into the predictors of visual outcomes. We found that the ELM_DL was likely to have a similar impact to the ONL_DL in terms of the contribution of the objective variable, which was eliminated because of multicollinearity (Table 3). It has been reported that the continuity of the outer retinal structure is correlated with the visual function, and that they were significantly correlated with the visual acuity prognosis [24,25,26,27]. We were able to confirm the similarity of the preoperative morphological information of the outer retina and the preoperative visual acuity.

On the other hand, (OPL_DL) − (ONL_DL) and (OPL_DL)/(ONL_DL) are combinations of distance information between layers and are novel features that have not been reported to contribute to the prognosis of the visual function. What these findings indicate is that the presence of a stenosis pattern with a narrowing from the OPL to the ONL is important for good postoperative visual function. In the previously reported DHI and HDR and stenotic MH, the morphology was reported to have a better anatomic prognosis [11,30]. However, the association with the visual function has not been reported in great detail.

The DHI was included in the features of the vector data combination, but it was not significantly related to the prediction of the visual function. Therefore, it is possible that we are the first to report that a stenosis of the MH is associated with the prognosis of the postoperative visual function.

The prognostic model created in this study is unique in that it was designed not only to improve the model’s accuracy, but also to be fit for the purpose of its contribution to improve the patient’s motivation. Assuming that the model is used in clinical practice, a vitrectomy for MH is the only method to improve vision, and it is necessary for patients who wish to improve their vision. Predicting the prognosis of the treatment is intended to motivate the patient, but it is a fact that not all patients have good postoperative vision. However, it is not a desirable prediction to reduce their motivation prior to treatment. Therefore, we believe that it is necessary to design an AI alignment that would contribute to improving the patient’s motivation as much as possible.

Therefore, we created a model with high accuracy and low recall by adjusting the ROC curve of the accuracy–recall curve. We left open the possibility that even if the visual acuity prognosis was classified to less than 0.7 decimal visual acuity units, we could present the possibility that the prediction could be off because of the low recall. Such a prognosis could potentially allow us to choose to intervene earlier than usual for such cases, and it is known from other cases reported that earlier surgical intervention is associated with a better prognosis [31].

The use of AI in medical decision making raises ethical concerns, particularly on patient autonomy and the transparency of algorithmic predictions. When using such AI tools in real-world clinical settings, it is essential to ensure that the AI tools are used to support rather than replace clinical judgments.

The limitations of this study include the use of standard regression coefficients, which do not represent the interactions among the explanatory variables. The standard regression coefficients for the (OPL_DL) − (ONL_DL) and (OPL_DL)/(ONL_DL) categories are related to the stenosis of the MH form that was found. Each of the ONL_DL and OPL_DL coefficients is a common term in the explanatory variables, and there may be an interaction between ONL_DL and OPL_DL. Further investigations of our methods to assess the interaction of these explanatory variables would allow a better interpretation of the results of the contributions of the explanatory variables.

The next limitation was that we annotated the B-scan image clippings by stratum, and the distance information was measured automatically by the program. Our program was unable to measure the height information of the MH in the feature values because it was difficult to define the height of the MH in many cases because the bottom of MH was not horizontal in the images. For this reason, the MHI and THI [32], which have been associated with a postoperative visual function prognosis in the past, could not be examined. Future research should focus on developing a method for measuring MH height, even when the bottom is tilted.

In this study, annotation was performed manually, which proved to be costly and time-consuming. To address this, we are currently conducting joint research aimed at automating the annotation process using advanced clarification technology. This automation is expected to enhance consistency and reduce the labor involved in data preparation.

## 5. Conclusions

Our results indicate that the morphology of the MH, especially a stenosis pattern narrowing from the OPL to the ONL, may be an important factor in predicting the 6-month postoperative visual acuity. Thus, it can be used to classify MH patients into groups with good or poor postoperative visual acuity.

Although only a small number of data were available, we could obtain a good predictive performance for the postoperative visual acuity classification while ensuring explainability by using ML.

## Figures and Tables

**Table 1:** Features established from the patients’ background information and features of the OCT images.

Features	Definition	Variable Types 1 = Categorical 2 = Continuous
Sex	Man or woman	1
Age	N/A	2
Preoperative BCVA	N/A	2
Method	vitrectomy or phacovitrectomy	1
Affected eye	Right or left	1
Axial length	N/A	2
Stage	N/A	1
Disease duration	N/A	2
ILM	Peel or invert or not peel	1
VMT	Present or absent of VMT	1
PVD	PVD complete or not complete	1
BDM	basal diameter of MH	2
Hole-min	minimum linear diameter of MH	2
OPL-DL	DL for OPL	2
ELM-DL	DL for ELM	2
EZ-DL	DL for EZ	2
Green-sDL	shortest DL for green color area	2
Yellow-sDL (NL-DL)	shortest DL for yellow color area	2
Sky blue-sDL	shortest DL for sky blue color area	2
Blue-sDL	shortest DL for blue color area	2
Area IRF	Area of IRF	2
Area-green	Area from ILM to OPL not including boundary line	2
Area-yellow	Area from OPL to ELM not including boundary line, ONL	2
Area-sky_blue	Area from ELM to EZ not including boundary line	2
Area-blue	Area from EZ to RPE not including boundary line	2
(OPL-DL) − (ONL-DL)	(OPL-DL) minus (ONL-DL)	2
(ELM-DL) − (ONL-DL)	(ELM-DL) minus (ONL-DL)	2
(OPL-DL) − (Green-sDL)	(OPL-DL) minus (Green-sDL)	2
(ELM-DL) − (Sky blue-sDL)	(ELM-DL) minus (Sky blue-sDL)	2
(EZ-DL) − (Sky blue-sDL)	(EZ-DL) minus (Sky blue-sDL)	2
(EZ-DL) − (Blue-sDL)	(EZ-DL) minus (blue-sDL)	2
BDM − (Blue-sDL)	BDM minus (blue-sDL)	2
BDM − (hole-min)	BDM minus (hole-min)	2
(Green-sDL)/(OPL-DL)	(Green-sDL) divide by (OPL-DL)	2
(OPL-DL)/(ONL-DL)	(OPL-DL) divide by (ONL-DL)	2
(ELM-DL)/(ONL-DL)	(ELM-DL) divide by (ONL-DL)	2
(ELM-DL)/(Sky blue-sDL)	(ELM-DL) divide by (Sky blue-sDL)	2
(EZ-DL)/(Sky blue-sDL)	(EZ-DL) divide by (Sky blue-sDL)	2
(EZ-DL)/(Blue-sDL)	(EZ-DL) divide by (Blue-sDL)	2
BDM/(Blue-sDL)	BDM divided by (Blue-sDL)	2
BDM/(Hole-min)	BDM divide by (Hole-min)	2

ILM = internal limiting membrane; VMT = vitreomacular traction; PVD = posterior vitreous detachment; MH = macular hole; OPL = outer plexiform layer; DL = defect length; ELM = external limiting membrane; EZ = ellipsoid zone; IRF = internal retinal fluid; sDL = shortest DL; ONL = outer nuclear layer; BCVA = best-corrected visual acuity; logMAR = logarithm of the minimum angle of resolution; Group A = postoperative BCVA < 0.15 logMAR units; and Group B = postoperative BCVA > 0.15 logMAR units.

**Table 2:** Baseline features of patients.

Variables	Group A	Group B	*p*-Value
Sex Men, n (%)	10 (30.3)	4 (36.4)	1.0
Age years	65.59 ± 6.51	67.18 ± 4.63	0.552
Preoperative BCVA	0.46 ± 0.24	0.81 ± 0.34	* 0.007
Methods PPV/phaco + PPV	1/31	0/11	1.0
Affected eye Right, n (%)	18 (54.5)	6 (36.0)	1.0
Axial length	23.98 ± 1.38	24.05 ± 1.56	0.987
Stage of MH (2, 3, 4)	3, 24, 5	3, 7, 1	0.524
Disease duration	1.72 ± 2.05	3.18 ± 3.46	0.481
ILM peeled, n (%)	22 (84.6)	4 (15.4)	0.147
VMT presence, n (%)	20 (76.9)	6 (23.1)	0.728
PVD exist, n (%)	27 (73.0)	10 (27.0)	1.0
BDM	724.66 ± 189.13	958.45 ± 399.37	0.162
Hole-min	295.50 ± 107.49	423.09 ± 175.49	0.053
OPL-DL	465.09 ± 140.14	492.36 ± 170.95	0.784
Yellow-sDL (ONL-DL)	313.28 ± 115.73	434.09 ± 186.22	0.015
ELM-DL	320.63 ± 120.25	480.82 ± 221.77	0.017
EZ-DL	359.00 ± 172.47	517.09 ± 263.87	0.217
Green-sDL	462.91 ± 134.37	533.73 ± 148.32	0.180
Sky blue-sDL	297.13 ± 120.35	458.27 ± 220.81	0.053
Blue-sDL	372.25 ± 168.61	530.82 ± 256.59	0.136
Area IRF	1437.72 ± 910.70	2240.36 ± 1653.83	0.109
Area-green	7268.78 ± 1414.01	7122.45 ± 1195.36	0.721
Area-yellow	5263.59 ± 1220.27	5068.27 ± 1463.88	0.799
Area-sky_blue	1585.31 ± 327.78	1560.64 ± 215.41	0.721
Area-blue	1701.56 ± 417.81	1744.73 ± 472.78	0.552
(OPL-DL) − (ONL-DL)	151.81 ± 114.31	58.27 ± 46.37	0.020
(ELM-DL) − (ONL-DL)	7.34 ± 42.69	46.73 ± 76.45	0.336
(OPL-DL) − (Green-sDL)	2.19 ± 95.99	−41.36 ± 55.85	0.362
(ELM-DL) − (Sky blue-sDL)	23.50 ± 30.58	22.55 ± 22.67	0.843
(EZ-DL) − (Sky blue-sDL)	61.88 ± 82.83	58.82 ± 51.32	0.516
(EZ-DL) − (Blue-sDL)	−13.25 ± 25.11	−13.73 ± 22.21	0.984
BDM-(Blue-sDL)	352.41 ± 127.58	427.64 ± 231.34	0.616
BDM-(hole-min)	429.16 ± 134.12	535.36 ± 250.01	0.616
(Green-sDL)/(OPL-DL)	1.01 ± 0.17	1.12 ± 0.17	0.362
(OPL-DL)/(ONL-DL)	1.63 ± 0.60	1.19 ± 0.19	0.020
(ELM-DL)/(ONL-DL)	1.03 ± 0.14	1.11 ± 0.16	0.336
(ELM-DL)/(Sky blue-sDL)	1.10 ± 0.14	1.05 ± 0.06	0.843
(EZ-DL)/(Sky blue-sDL)	1.20 ± 0.23	1.11 ± 0.09	0.516
(EZ-DL)/(Blue-sDL)	0.95 ± 0.08	0.96 ± 0.05	0.984
BDM/(Blue-sL)	2.19 ± 0.81	1.92 ± 0.41	0.784
BDM/(Hole-min)	2.62 ± 0.75	2.33 ± 0.43	0.267

BCVA = best-corrected visual acuity; PPV = pars plana vitrectomy; phaco, phacoemulsification cataract surgery; MH = macular hole; ILM = internal limiting membrane; VMT = vitreomacular traction; PVD = posterior vitreous detachment; OPL = outer plexiform layer; DL = defect length; ELM = external limiting membrane; EZ = ellipsoid zone; IRF = internal retinal fluid; sDL = shortest DL; ONL = outer nuclear layer. Group A is postoperative BCVA < 0.7 and Group B is postoperative BCVA ≥ 0.7. *p* values were calculated by comparing each variable between Group A and Group B. * Significant at *p* < 0.05 (the Kolmogorov–Smirnov test was used for the continuous quantity variables. Fisher’s exact test was used as the categorical variable).

**Table 3:** Selection of explanatory variables.

Variables	1st R^2^ Score	2nd R^2^ Score
Preoperative BCVA	0.321	0.283
ELM DL	0.919	NA
ONL DL	0.918	0.521
(OPL DL) − (ONL DL)	0.722	0.660
(OPL DL)/(ONL DL)	0.767	0.755

**Table 4:** Results of classification accuracy.

Algorithm	Mean	Standard Deviation	95% Confidence Interval for Mean
Accuracy	0.738	0.130	0.712~0.764
Precision	0.921	0.088	0.903~0.938
Recall	0.734	0.162	0.702~0.766
F-measure	0.804	0.116	0.781~0.827
AUC	0.843	0.117	0.820~0.866

**Table 5:** Coefficients of explanatory variables.

Variables	Mean	Standard Deviation	95% Confidence Interval for Mean
Preoperative BCVA	0.281	0.028	0.275~0.286
ONL DL	0.130	0.038	0.123~0.138
(OPL DL)/(ONL DL)	−0.174	0.020	−0.178~−0.170
(OPL DL) − (ONL DL)	−0.212	0.029	−0.217~−0.206

## Data Availability

Data are unavailable due to privacy or ethical restrictions.
